# Simultaneous Determination of Pesticide Residues and Mycotoxins in Storage Pu-erh Tea Using Ultra-High-Performance Liquid Chromatography Coupled with Tandem Mass Spectrometry

**DOI:** 10.3390/molecules28196883

**Published:** 2023-09-30

**Authors:** Siu Leung Chau, Aihua Zhao, Wei Jia, Lu Wang

**Affiliations:** 1 School of Chinese Medicine, Hong Kong Baptist University, Kowloon Tong, Hong Kong, China; slchau@hkbu.edu.hk (S.L.C.); weijia1@hkbu.edu.hk (W.J.); 2 Shanghai Sixth People’s Hospital Affiliated to Shanghai Jiao Tong University School of Medicine, Shanghai 200023, China; zhah@sjtu.edu.cn

**Keywords:** Pu-erh tea, pesticide residues, mycotoxins, UPLC-MS/MS, QuEChERS, method validation

## Abstract

Mycotoxins and pesticides are the most concerning chemical contaminants that can affect the quality of Pu-erh tea during its production and storage. This study presents a method that can simultaneously determine 31 pesticide residues and six mycotoxins in Pu-erh tea within 11 min using ultra-high-performance liquid chromatography coupled with tandem mass spectrometry (UPLC-MS/MS) after QuEChERS extraction. The lower limit of quantification (LOQ) for all analytes ranged between 0.06 and 50 ppb. Recoveries for each pesticide and mycotoxin ranged between 62.0 and 130.3%, with intra- and inter-day precisions lower than 15%. Good linear relationships were obtained, with correlation coefficients of r^2^ > 0.991 for all analytes. The established method was applied to 31 Pu-erh tea samples, including raw and ripened Pu-erh tea with different storage times. As a result, pesticide residues were not detected in any of the collected samples, and the mycotoxins detected in the samples were well below the official maximum residue limits (MRLs). Notably, the levels of aflatoxin B_1_ (AFB1), aflatoxin G_1_ (AFG1) and aflatoxin G_2_ (AFG2) were lower than 1 ppb in the samples stored for more than 30 years.

## 1. Introduction

Pu-erh tea, documented for thousands of years since the Tang dynasty in China, is well-known for its exceptional flavor and taste [1]. Depending on whether it undergoes a fermentation procedure, Pu-erh tea is distinguished into raw Pu-erh tea and ripened Pu-erh tea [2]. The consumption of Pu-erh tea has rapidly increased worldwide due to its various beneficial effects on human health, such as attenuating hypercholesterolemia and having antiobesity, antiaging (by modulating gut microbiota and bile acid metabolism) [2,3,4,5], anticancer [6], anti-inflammation [7], antidiabetic [8,9,10], neuroprotective [11], hypolipidemic and antioxidant properties [12,13,14]. Additionally, the flavor of Pu-erh tea tends to improve with increased storage time [15]. Thus, it has been widely accepted that the longer Pu-erh tea is stored, the higher its market price [16]. As the number of consumers is continuously increasing, monitoring harmful contaminants such as pesticide residues and mycotoxins in Pu-erh tea has become essential for ensuring public health safety [17].

Pu-erh tea production takes a long time, including planting, primary and refined processing, packaging, storage and transportation for final consumption [18]. Pesticides are frequently employed to increase harvest yields to meet market demands. However, the high concentration of pesticide residues in Pu-erh tea may pose a risk to humans [19]. Long-term exposure to pesticides can lead to chronic diseases like cancer, birth defects, reproductive harm, neurological damage and disruption of the endocrine system [20], raising significant concerns about environmental safety and human health. Hence, many countries and organizations have established criteria for maximum residue limits (MRLs) for tea to regulate the international tea trade.

Mycotoxins, natural toxins produced by fungi, pose another concern in tea samples. The long-term post-fermentation process and storage of Pu-erh tea can promote fungal growth, thereby increasing the potential risk of mycotoxin contamination [21]. Mycotoxins exhibit various harmful properties, including genotoxicity, carcinogenicity, immunosuppressive activity, mutagenicity, nephrotoxicity and teratogenicity [22]. Aflatoxins, one of the most potent health hazards found in tea, are prevalent in China and India, two of the largest tea-producing countries, posing a significant risk for aflatoxin exposure [23]. Nearly 18 different types of aflatoxins have been identified, among which AFB1 is the most toxic and generally found in the highest quantities. Although AFB1 is classified as a class 1 human carcinogen by the International Agency for Research on Cancer, only a few studies have investigated mycotoxin contamination in post-fermented tea [24].

Due to the relatively low quantities of pesticide residues and mycotoxins in tea, extraction and enrichment are necessary before analysis. Commonly used techniques for extracting mycotoxins from tea include liquid–liquid extraction (LLE), solid-phase extraction (SPE) and dispersive liquid–liquid microextraction (DLLME) [23]. QuEChERS (Quick, Easy, Cheap, Effective, Rugged and Safe) is a new enrichment method based on dispersive solid-phase extraction (d-SPE). It has been developed and widely used to extract multiple pesticides from tea [25] and mycotoxins from food and liquid samples [26]. Therefore, QuEChERS would be a suitable choice for extracting both pesticides and mycotoxins from Pu-erh tea, since it would help minimize the sample treatment procedure and prevent exposure to matrix effects.

Currently, the most employed techniques for analyzing pesticides and mycotoxins are, respectively, gas chromatography–mass spectrometry (GC-MS) and liquid chromatography–tandem mass spectrometry (LC-MS/MS) [18,19,21,23,24,25,26,27,28,29,30,31,32,33,34]. Depending on the chemical properties of the pesticides and mycotoxins, these methods employ different extraction solvents, comprehensive cleanup steps and longer analysis times in GC or LC, aiming to reduce matrix effects and improve method sensitivity. However, there have been no reports about the simultaneous determination of pesticides and mycotoxins in tea.

In this study, we used ultra-high-performance liquid chromatography coupled with tandem mass spectrometry (UPLC-MS/MS) to simultaneously quantify the pesticide residues and mycotoxins after QuEChERS extraction. We collected 31 raw and ripened Pu-erh teas sourced from China from 1900 to 2021. Among them were two rare vintage raw Pu-erh teas (“1900 Song Ping Hao” and “1920 Jing Chang Hao”, produced in 1900 and 1920, respectively) that had been carefully stored for a century. As far as we know, most studies have focused on quantitatively determining mycotoxins or pesticide residues in relatively short-storage teas (less than ten years). There is currently no research about pesticide residues or mycotoxins in Pu-erh tea stored for an extended period. We aimed to: (a) develop a UPLC-MS/MS method to simultaneously analyze 31 pesticide residues and six mycotoxins; and (b) validate the method with 31 Pu-erh teas and present the levels of pesticide residues and mycotoxins in these Pu-erh tea samples.

## 2. Results and Discussion

### 2.1. Optimization of UPLC-MS/MS Conditions

Due to its sensitivity and selectivity for trace analysis of complex extracts, LC-ESI-MS/MS is currently the most commonly used technique for analyzing pesticide residues and mycotoxins using multiple reaction monitoring (MRM) [33]. To ensure maximum sensitivity for quantifying the target compounds, we optimized all MS parameters for each analyte. The MS/MS conditions were optimized in positive and negative ESI modes, including the optimization of precursor ions, product ions and collision energies. The most intense transition (*m*/*z*) was selected for quantitation, while another transition for each compound was chosen as a validated ion for identifying analytes in the quantitative analysis. The optimized cone and collision voltages (CV and CE, respectively) for the quantitative and qualitative transition ions are summarized in Table 1.

**Figure 1:**
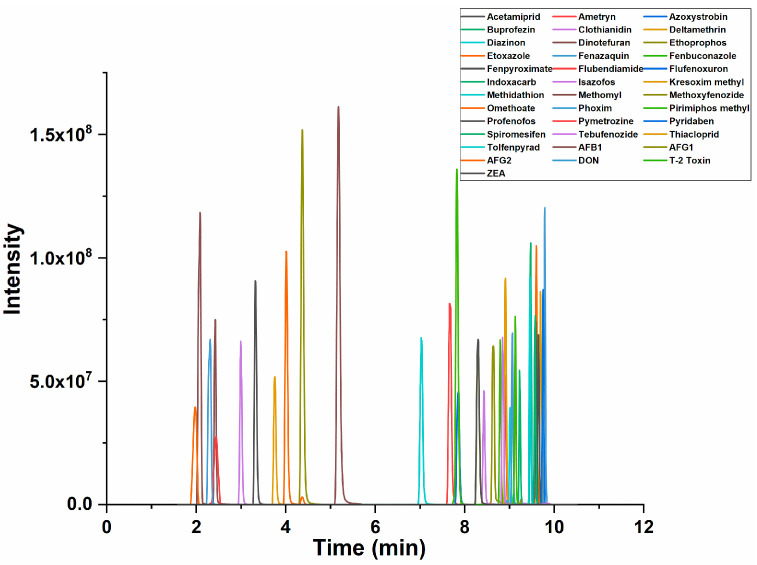
Total ion chromatogram of 31 pesticide residues and 6 mycotoxins.

To achieve the best peak shape, higher sensitivity and shorter analysis time, an optimization of the chromatographic conditions was carried out. Methanol and acetonitrile were tested as organic phases, both showing good peak shapes. Acetonitrile was selected due to the shorter analysis time. Formic acid, acetic acid and ammonium acetate (5.0, 10.0 and 15.0 mM, respectively) were evaluated as mobile-phase modifiers. It was found that 0.1% of formic acid provided the same sensitivity and resolution as 5 mM of ammonium acetate for mycotoxins (AFB1 was taken as an example; the MRM chromatograms under different mobile-phase conditions are shown in Figure S1A). For pesticides, the highest peak intensity was observed under 5 mM of ammonium acetate for most pesticides (fenazaquin was taken as an example; the MRM chromatograms under different mobile-phase conditions are shown in Figure S1B). A few pesticide residues, such as pirimiphos methyl and tolfenpyrad, indicated that 10 mM of ammonium acetate might be optimal, but the intensities were not significantly increased compared to 5 mM. Moreover, the effect of 10 mM of ammonium acetate on the chromatographic performance was found to be deleterious, greatly increasing the back pressure on the column [25]. Consequently, 5 mM of ammonium acetate was chosen as the mobile phase. The typical UPLC-MS/MS total ion current (TIC) chromatogram of 31 pesticide residues and six mycotoxins under optimized conditions is shown in Figure 1. The retention times of each compound are listed in Table 1, and the MRM chromatograms of each compound are shown in Figure S3.

### 2.2. Method Validation

In the validation method, before spiking the analytical portions with the required amount of pesticide and mycotoxin mixtures, the blank samples were tested and checked for the absence of any of the target pesticides and mycotoxins. The validation was performed with blank Pu-erh tea, standards in the solvent and standards in the matrix extracts obtained according to the selected sample treatment. The specific procedures for determining the different validation parameters are summarized in the following sections.

For each pesticide and mycotoxin, the selectivity, precision, accuracy, linearity and limit of quantification (LOQ) were calculated to validate the analytical method used in this study.

#### 2.2.1. Selectivity

Identifying the target compounds from potentially interfering substances can be performed by analyzing their two MRM transitions, their typical retention times and their relative abundances. Figures S2 and S3 show the representative MRM chromatography of the blank and spiked tea samples, respectively. No interference of the analytes was observed, and all the analytes could be well defined by their MRM transitions and retention times.

#### 2.2.2. Linearity and LOQ

Under the optimum analysis conditions, linearity was studied at eight different level points by matrix-matched standard calibration. The concentrations of the calibration levels were between 0.06 and 1000 ppb. The linear ranges and regression coefficients (r^2^) are summarized in Table 1, and all the regression coefficients (r^2^) are higher than 0.991.

The LOQ was calculated by injecting lower pesticide concentrations (in the spiked blank tea extracts) that yielded an S/N equal to or higher than 10. The LOQs are summarized in Table 1. The LOQs were equal to or lower than the MRLs following the National Standard Maximum Residue Limits of Pesticides in Food (GB 2763-2021) and China’s Maximum Levels for Mycotoxins in Foods (GB 2761-2017). The method is particularly favorable for analyzing pesticides and mycotoxins in tea.

#### 2.2.3. Intra- and Inter-Day Precision

The precision of the extraction method was determined by calculating the average of three spiked blank matrices analyzed at three different concentration levels (low, medium and high). The precision of the chromatographic method (represented as the relative standard deviation, RSD%) was obtained from six replicates. Precision is expressed as the RSD (%) of the intra-day and inter-day analyses over three consecutive days. In Table 2, the intra- and inter-day RSDs are lower than 15% for all pesticides and mycotoxins, which is consistent with the current European legislation (%RSD ≤ 20%).

#### 2.2.4. Matrix Effect and Recovery

Matrix-matched calibrations were prepared in the extracted blank Pu-erh tea as the representative matrix and in the solvent. The effect of the matrix was investigated in terms of the co-elution of the sample matrix components at the same retention time with the target analyte, which can result in ion suppression or enhancement. Matrix-matched standards were prepared by spiking the standard solution at three concentration levels (low, medium and high). The peak areas obtained from the quantitative transition ions were compared to those obtained from a solvent standard at an equivalent concentration. The matrix effects were calculated with the equation:

Matrix effect = [(Area in matrix)/(Area in solvent) − 1] × 100%

The detailed values of the matrix effects are shown in Table 2. The matrix effect can be considered weak if it is within ±20%, whereas exceeding the scope of ±20% indicates significant matrix enhancement or suppression. In the current method, most of the analytes exhibited matrix suppression, as the matrix components decreased the efficiency of droplet formation and/or decreased the number of ions formed in LC-MS [35].

The recovery of the method was studied by calculating the recoveries of the analytes using the standard addition. The reference standards were added at three different concentration levels (low, medium and high), with six parallels at each level. The solutions were prepared according to the sample preparation procedure. The recoveries of the pesticide residues and mycotoxins were tested with a blank Pu-erh tea extract as the matrix. The results of the recoveries are presented in Table 2, in which only the recoveries of DON in the medium and high levels were outside the preferred range of 70% to 110% but were still accepted for quantification in this study [36].

### 2.3. Pu-erh Tea Sample Analysis

The applicability of the proposed method was assessed for the analysis of 31 Pu-erh tea samples, whose information is summarized in Table 3. The quantitative results revealed that no pesticide residues were detected in any of the samples. Regarding mycotoxins, AFB1, AFG1, AFG2 and DON were detectable in 12 out of the 23 raw Pu-erh teas, in which the levels of AFB1, AFG1 and AFG2 were all lower than 1.0 ppb (each 5 ppb MRL) in 5 teas stored for 30 (including 2 originals), 42, 72 and 102 years, respectively, and the levels of DON were lower than 70 ppb (1000 ppb MRL) in 7 teas stored for less than 12 years. Additionally, 4 out of the 31 Pu-erh teas, including 1 raw tea and 3 ripened teas, were detected to have T-2 toxins, with all levels below 60 ppb (200 ppb MRL).

Contamination with Aspergillus flavus and the subsequent production of aflatoxins present one of the most severe safety problems worldwide. Studies have shown that aflatoxin levels can exceed the 20 ppb FDA/WHO regulatory limits with storage time in maize samples stored for more than six months [37]. In the present study, we detected AFB1, AFG1 and AFG2 in raw Pu-erh tea samples with long storage years (>30 years). However, these aflatoxins were lower than 1 ppb, well below the FDA/WHO regulations or China’s Maximum Levels for Mycotoxins in Foods (GB 2761-2017). An explanation for finding fungi capable of producing mycotoxins but not detecting the toxins themselves (or at a very low level) may be found in a recent report that stated that tea extracts inhibited aflatoxin production by *Aspergillus flavus*, although they did not inhibit the mycelial growth of the fungus itself. Raw Pu-er tea and ripened Pu-erh tea extracts were found to reduce aflatoxin production by nearly 90% [38]. Another finding is that the antioxidant gallic acid inhibits aflatoxin formation and growth in *Aspergillus flavus* in a dose-dependent manner [39]. During the pile-fermentation process of ripened Pu-erh tea, the level of gallic acid increased more than 11 times. The high levels of gallic acid could inhibit aflatoxin generation, which may partly explain why we did not detect aflatoxins in the freshly ripened Pu-erh teas.

Deoxynivalenol (DON) is primarily produced by *Fusarium graminearum* and *Fusarium culmorum*. The ideal conditions for DON are water activity <0.9 under 25 °C [40]. In our study, DON was lower than 70 ppb, much lower than the 1000 ppb criterion in China’s Maximum Levels for Mycotoxins in Foods (GB 2761-2017). It has been reported that fungicides can stimulate DON’s production, especially in low doses, as ineffective doses promote mild-to-medium stress levels for the fungus [41]. Recently, fungicides have been used as a complementary control measure when weather conditions are conducive to infection from anthesis to harvest. This may explain why low levels of DON were identified in the fresh raw Pu-erh tea samples but not detected in the long-term stored Pu-erh tea. We also found that DON was not detected in the ripened Pu-erh tea. DON is water-soluble, and some evidence indicates that DON levels may be reduced during processing, mainly boiling in water [42]. In the pile-fermentation process of ripened Pu-erh tea, the high temperature and moisture may further decrease DON concentration. Additionally, the changes in microbial communities during pile fermentation, with *Aspergillus* being the dominant fungus and low levels of *Fusarium graminearum* and *Fusarium culmorum*, could lead to poor DON products in the final stage of fermentation [43].

## 3. Materials and Methods

### 3.1. Samples of Pu-erh Tea

Thirty-one different samples of Pu-erh tea were provided by the International Pu-erh Tea Association and Yunnan Pu-erh Tea Factory Co., Ltd (Pu-erh, China). These samples were produced in Yunnan Province, which is the main production area of Pu-erh tea, as detailed in Table 3. The samples were kept away from light and moisture at room temperature until analysis. Three batches of market Pu-erh tea samples were used as a blank matrix in this study.

### 3.2. Chemicals, Materials and Standards

Thirty-one pesticide standards with purity ≥ 98%, including ametryn, azoxystrobin, buprofezin, clothianidin, deltamethrin, diazinon, dinotefuran, ethoprophos, etoxazole, fenazaquin, fenbuconazole, flubendiamide, flufenoxuron, indoxacarb, isazofos, kresoxim methyl, methidathion, methomyl, methoxyfenozide, omethoate, phoxim, pirimiphos methyl, profenofos, pymetrozine, pyridaben, spiromesifen, tebufenozide, thiacloprid and tolfenpyrad, were purchased from J&K Scientific Ltd. (Beijing, China). Six standards of mycotoxin with purity ≥98%, including AFB1, AFG1, AFG2, DON, T-2 toxin and ZEA, were purchased from Genetimes Excell International Holdings Limited. Acetamiprid and fenpyroximate with purity >98% were purchased from Dieckmann (Hong Kong, China) Chemical Industry Company Limited. LC-MS-grade solvents, including methanol, acetonitrile, formic acid, acetic acid and ammonium acetate, were purchased from Sigma-Aldrich (St. Louis, MO, USA). Ultrapure water was prepared by the Millipore purification system (Avidity Science, UK). QuEChERS was obtained from Waters Corp., Milford, MA, USA, for sample preparation.

### 3.3. Preparation of Standard Solutions

A mixture of 31 certified pesticides and 6 mycotoxins was used. The individual stock solution of each standard (0.1 mg/mL) was prepared in methanol and stored at −20 °C. The working standard solutions were prepared by diluting the corresponding stock solution with methanol. The mixed stock solution was prepared in methanol at 20 µg/mL, and the working standard solutions were prepared by serial dilutions with eight levels of concentration. The obtained solutions were stored in a refrigerator at 4 °C. The matrix-matched standards with similar concentrations were prepared by using the blank sample matrix extract.

### 3.4. Sample Preparation

The extraction of pesticide residues and mycotoxins was performed according to the QuEChERS sample preparation protocol. The tea sample was ground into powder and sieved through a 425 µm sieve. A total of 100 mg of ground tea was accurately weighed and transferred to a 2.0 mL Eppendorf Safe-Lock tube. A total of 1.5 mL of acetonitrile/water (50/50) and 350 mg DisQuE™ powder were added to the tube. The mixture was then shaken vigorously for 1 min and then subsequently centrifuged at 12,000 rpm for 10 min. An aliquot of 0.6 mL of the supernatant was transferred into a 2 mL dispersive solid-phase extraction (d-SPE) tube. This tube contained 150 mg of MgSO4 to remove water from the organic phase, 50 mg of primary secondary amine (PSA) to eliminate various polar organic acids, polar pigments, some sugars and fatty acids and 50 mg of graphitized carbon black (GCB) to remove sterols and pigments. After that, the mixture was shaken vigorously for 1 min and centrifuged at 12,000 rpm for 10 min. A total of 0.1 mL of the supernatant was transferred to a 1.5 mL centrifuge tube and diluted to 0.2 mL with a 50/50 acetonitrile/water mixture, then transferred into a glass vial for analysis using UPLC-MS/MS.

### 3.5. UPLC-MS/MS Analysis

The quantification of pesticide residues and mycotoxins was performed using an ultra-high-performance liquid chromatography coupled with a tandem mass spectrometry (UPLC-MS/MS) system (ACQUITY UPLC-Xevo TQXS, Waters Corp., Milford, MA, USA). Chromatographic separations were performed on a BEH C18 column (100 mm × 2.1 mm id, 1.7 μm, Waters, Milford, MA, USA) at 40 °C with elute A (water with 5 mM of ammonium acetate) and elute B (acetonitrile). The gradient was as follows: 0–0.2 min, 2% B; 0.2–1.5 min, 2–30% B; 1.5–3 min, 30–40% B; 3–4.5 min, 40-45% B; 4.5–7.5 min, 45–65% B; 7.5–9 min, 65–98% B; 9.0–11.0 min, 98% B. The flow rate was 0.4 mL/min. The mass spectrometer was equipped with an ESI source and operated in positive- and negative-ion modes. The source settings were as follows: the capillary voltage was set at 1.5 kV, the desolvation temperature was set at 500 °C, the desolvation gas flow was set at 1000 L/h and the cone gas flow rate was set at 150 L·h^−1^.

### 3.6. Identification and Quantification of the Pesticide Residue

The identity of pesticide residue and mycotoxins in an extract was confirmed by its retention time matching that of the corresponding analyte in the pure standard solutions and the appearance of two product ion transitions that matched the relative intensity criteria specified by the EU SANTE/12682/2019 guidelines. Once the analyte was confirmed in an extract, the concentration of the analyte was calculated by the corresponding calibration function.

### 3.7. Data Processing

The main peak areas in each chromatogram were quantified by setting up the Masslynx 4.2 processing software (Waters Corp., Milford, MA, USA).

## 4. Conclusions

In this study, an efficient and rapid method for simultaneously determining 31 pesticide residues and six mycotoxins was established and validated using UPLC-MS/MS after QuEChERS extraction. The validation of the method indicated that the LOQs were lower than or equal to the MRLs established by the National Food Safety Standard. The developed method was applied to determine pesticide residues and mycotoxins in long-term storage Pu-erh tea. The results revealed that the levels of all analytes remained within or below the MRLs established by the National Food Safety Standard. Notably, Pu-erh tea from ‘1900 Song Ping Hao’ and ‘1920 Jing Chang Hao’, which had been stored for more than 100 years, did not show any detection of pesticide residues, and the mycotoxins detected remained below the MRLs.

## Figures and Tables

**Table 1:** MRM parameters, retention times, linear range, regression coefficients (r^2^), LOQ and MRL of the target compounds.

Type of Analytes	Compounds	ESI mode	RT (min)	Precursor Ion (*m*/*z*)	Cone (V)	Quantification Ion		Qualification Ion		Linearity	Linear Range	LOQ	MRL
						CE (eV)	Product (*m*/*z*)	CE (eV)	Product (*m*/*z*)	r^2^	(ppb)		
**Pesticide**	Acetamiprid	+	3.3	223.2	25	20	56.0	15	126.0	0.997	0.13–100	0.13	10 ^a^
	Ametryn	+	7.7	228.2	25	20	68.0	15	186.0	0.998	1–1000	1	50 ^a^
	Azoxystrobin	+	7.8	404.0	25	15	344.1	14	372.0	0.994	5–1000	5	10 ^a^
	Buprofezin	+	9.5	306.2	30	16	116.1	12	201.0	0.998	0.25–1000	0.25	10 ^a^
	Clothianidin	+	3.0	250.2	30	15	132.0	10	169.1	0.997	0.13–100	0.13	10 ^a^
	Deltamethrin	+	9.7	505.9	35	17	280.9	15	328.3	0.997	5–1000	5	10 ^a^
	Diazinon	+	9.0	305.0	25	20	153.2	15	169.1	0.999	0.13–100	0.13	10 ^a^
	Dinotefuran	+	2.1	203.1	30	10	113.0	10	129.2	0.996	0.13–100	0.13	20 ^a^
	Ethoprophos	+	8.6	243.1	33	19	130.9	27	97.0	0.996	0.06–1001	0.06	10 ^a^
	Etoxazole	+	9.6	360.2	55	55	113.1	30	141.0	0.996	0.25–1000	0.25	10 ^a^
	Fenazaquin	+	9.8	307.2	25	15	57.1	14	161.2	0.995	5–1000	5	300 ^a^
	Fenbuconazole	+	8.8	337.1	35	17	70.0	15	125.0	0.993	0.25–1000	0.25	10 ^a^
	Fenpyroximate	+	9.7	422.0	42	33	138.1	13	366.0	0.993	10–1000	10	50 ^a^
	Flubendiamide	+	8.9	683.0	25	20	273.9	13	408.0	0.996	10–1000	10	10 ^a^
	Flufenoxuron	+	9.6	489.0	25	20	141.1	15	158.1	0.994	10–1000	10	10 ^a^
	Indoxacarb	+	9.2	528.0	25	23	150.0	15	218.0	0.996	1–1000	1	20 ^a^
	Isazofos	+	8.4	314.1	25	15	120.0	8	162.1	0.994	0.25–100	0.25	10 ^a^
	Kresoxim methyl	+	8.9	314.1	15	15	222.2	14	235.0	0.998	0.25–100	0.25	10 ^a^
	Methidathion	+	7.0	303.0	15	20	85.0	15	145.1	0.991	0.5–100	0.5	1 ^a^
	Methomyl	+	2.4	163.2	20	8	88.0	8	103.0	0.996	0.13–100	0.13	20 ^a^
	Methoxyfenozide	+	8.3	313.0	25	15	133.0	14	149.0	0.998	0.5–100	0.5	20 ^a^
	Omethoate	+	2.0	214.1	15	15	183.0	11	196.0	0.998	0.25–100	0.25	10 ^a^
	Phoxim	+	9.1	299.0	25	15	77.0	14	129.0	0.995	0.13–100	0.13	50 ^a^
	Pirimiphos methyl	+	9.1	306.2	25	20	108.1	15	164.0	0.993	0.5–100	0.5	10 ^a^
	Profenofos	+	9.4	372.9	25	15	302.7	14	344.9	0.993	5–1000	5	10 ^a^
	Pymetrozine	+	2.5	218.0	30	17	105.0	15	107.0	0.997	0.13–100	0.13	20 ^a^
	Pyridaben	+	9.8	365.1	38	24	147.0	11	309.1	0.996	1–1000	1	100 ^a^
	Spiromesifen	+	9.6	393.0	45	17	295.2	15	303.3	0.991	0.5–100	0.5	15 ^a^
	Tebufenozide	+	8.9	353.2	30	10	133.1	20	105.1	0.998	0.25–100	0.25	10 ^a^
	Thiacloprid	+	3.8	253.1	45	21	126.0	36	90.0	0.993	0.13–100	0.13	20 ^a^
	Tolfenpyrad	+	9.5	384.2	25	29	171.1	15	197.0	0.992	10–1000	10	10 ^a^
**Mycotoxin**	AFB1	+	5.2	313.1	40	25	285.3	30	241.8	0.995	0.5–100	0.5	5 ^b^
	AFG1	+	4.4	329.1	40	30	243.4	30	215.0	0.999	0.5–100	0.5	5 ^b^
	AFG2	+	4.0	331.1	40	30	245.4	35	189.4	0.999	0.5–100	0.5	5 ^b^
	DON	+	2.3	297.1	40	15	203.5	15	231.4	0.995	50–1000	50	1000 ^b^
	T-2 Toxin	+	7.8	467.3	40	10	305.5	10	245.5	0.991	50–1000	50	200 ^c^
	ZEA	-	8.3	317.2	40	25	175.2	30	131.0	0.994	10–1000	10	60 ^b^

AFB1: aflatoxin B_1_; AFG1: aflatoxin G_1_; AFG2: aflatoxin G_2_; DON: deoxynivalenol; ZEA: zearalenone; RT: retention time; MRL: maximum residue limit; CE: collision energy; LOQ: limit of quantification; ^a^: National Food Safety Standard—MRL for Pesticides in Food (GB 2763-2021); ^b^: China’s MRL for Mycotoxins in Foods (GB 2761-2017); ^c^: MRL of contaminants in foods in the EU (EC Regulation No 881/2006); +: positive mode; -: negative mode.

**Table 2:** Intra- and inter-day precision, recoveries and matrix effects.

Type of Analytes	Compounds	Repeatability (RSD %)		Spiking Level								
				Low			Medium			High		
		Intra-Day	Inter-Day	Recovery (%)	RSD (*n* = 6)	Matrix Effects	Recovery (%)	RSD (*n* = 6)	Matrix Effects	Recovery (%)	RSD (*n* = 6)	Matrix Effects
**Pesticide**	Acetamiprid	7.4	12.3	73.5	8.5	0.1	85.5	8.6	1.0	111.3	18.8	−5.0
	Ametryn	7.6	8.8	94.1	22.6	11.0	87.2	8.3	10.0	87.7	5.7	−1.0
	Azoxystrobin	7.8	11.1	85	5.4	−4.0	92.9	10.5	−1.0	89.4	13.9	−3.0
	Buprofezin	8.6	7.5	91.4	10.9	4.0	94.7	4.8	3.0	77.5	9.3	−7.0
	Clothianidin	5.3	12.0	85.4	20.1	28.0	75.8	10.1	20.0	74.8	17.2	2.0
	Deltamethrin	2.8	6.4	94.1	4.1	11.0	89.7	7	11.0	83	5.1	−15.0
	Diazinon	5.9	3.2	97.7	3.4	−1.0	100.2	11.1	0.0	80.4	7.1	−5.0
	Dinotefuran	2.8	6.4	114.4	26.1	7.0	106	12.8	6.0	84.5	11.1	4.0
	Ethoprophos	2.5	3.5	94.2	4.2	3.0	103.5	9.2	1.0	93.7	5.4	−3.0
	Etoxazole	7.3	7.9	104.4	17.6	5.0	96.8	14.2	1.0	97.8	16	−10.0
	Fenazaquin	10.5	13.9	97.5	1.9	−9.0	94.8	10.5	5.0	110.5	5.6	−22.0
	Fenbuconazole	3.2	7.4	101.6	5.7	0.0	105.6	10.4	0.0	84.1	10.6	−3.0
	Fenpyroximate	4.7	6.9	130.3	4.8	−32.0	129	10.7	−10.0	92.7	18.6	−22.0
	Flubendiamide	8.4	5.6	124.5	8.9	−30.0	114.6	9.2	−34.0	82	4.4	−4.0
	Flufenoxuron	5.0	8.3	105.1	0.6	−13.0	93.2	2.6	17.0	102.5	3.4	−14.0
	Indoxacarb	8.6	6.6	111	8.3	−29.0	112	4.6	−13.0	90.7	17.8	−4.0
	Isazofos	4.3	5.2	95.5	6.2	−6.0	100.5	7.8	0.0	96.7	0.8	−3.0
	Kresoxim methyl	1.8	8.6	94.1	3.5	−18.0	102.9	13	−15.0	98.6	11	1.0
	Methidathion	1.2	11.2	99.2	2.3	−10.0	100.1	12.7	−7.0	95.5	2.6	−3.0
	Methomyl	2.5	7.6	88.6	3.2	6.3	100	12.4	4.7	94.4	9.8	2.0
	Methoxyfenozide	4.4	2.5	95.1	4.2	−10.0	106.6	10.1	−3.0	82.8	1.4	−5.0
	Omethoate	1.9	7.9	87.4	6.1	4.0	92.4	9.8	5.0	79.5	2.7	0.0
	Phoxim	3.6	7.1	102.2	6.6	−25.0	98.8	1.1	−6.0	94.7	12.8	−22.0
	Pirimiphos methyl	6.1	6.5	114.5	1.2	−16.0	106.7	12.1	−6.0	107.3	12.8	−5.0
	Profenofos	9.0	6.0	104	1.9	−31.0	103.9	14.5	2.0	96.4	14.2	−17.0
	Pyridaben	5.5	13.5	81.0	8.4	23.0	82.7	14.1	15.0	88	15.5	−5.0
	Pyriproxyfen	8.6	6.7	112.9	4.2	2.0	99.1	11.9	0.0	95.3	12.9	−3.0
	Spiromesifen	3.4	6.4	89.8	7.9	14.0	100.5	10.1	3.0	99.2	19.7	−4.0
	Tebufenozide	3.9	7.5	90.5	6.5	0.0	104.9	10.9	1.0	97.5	1.5	−1.0
	Thiacloprid	2.4	12.3	99.4	16.8	10.0	92.5	12.8	5.0	97.4	19.8	−2.0
	Tolfenpyrad	5.4	8.0	102.7	1.7	−8.0	109	9.7	−5.0	101.7	16.5	−14.0
**Mycotoxin**	AFB1	5.2	12.8	112.9	14.2	−43.3	100.9	9.5	−41.1	112.7	3.3	−41.1
	AFG1	2.1	13.8	110.3	0.2	−40.2	108.9	0.6	−43.8	113.2	4.3	−47.7
	AFG2	2.6	12.8	96.8	0.2	−29.9	107.9	11.1	−42.4	101.2	5.6	−44.2
	DON	6.9	13.9	79.2	12.9	14.3	62	10.6	−12.3	65.8	17.4	−16.5
	T-2 Toxin	1.4	13.2	91.3	7.8	−1.3	70.6	11.7	2.8	83.6	12.2	−13.7
	ZEA	7.1	4.7	113.7	4.3	−39.8	121.8	2	−2.1	91.7	9.1	−7.3

AFB1: aflatoxin B_1_; AFG1: aflatoxin G_1_; AFG2: aflatoxin G_2_; DON: deoxynivalenol; ZEA: zearalenone.

**Table 3:** Information on all Pu-erh tea samples and concentration (ppb) of mycotoxins detected in each Pu-erh tea sample.

Brand	Year of Production	Type	Storage Duration	Origin (Yunnan Province)	AFB1	AFG1	AFG2	T-2 Toxin	DON
Bu Lang Shan	2021	Raw	1	Bulang mountain	ND	ND	ND	ND	61.8
Mi Di	2021	Raw	1	Mojiang County	ND	ND	ND	ND	54.9
Bu Lang Shan	2020	Raw	2	Bulang mountain	ND	ND	ND	ND	55.1
Nan Nuo	2020	Raw	2	Nannuo mountain	ND	ND	ND	ND	56.5
Meng Song	2020	Raw	2	Mengsong	ND	ND	ND	ND	55.5
Bu Lang Shan	2016	Raw	6	Bulang mountain	ND	ND	ND	ND	40.5
Wang Rui	2015	Raw	7	Unknown	ND	ND	ND	ND	ND
Bu Lang Shan	2011	Raw	11	Bulang mountain	ND	ND	ND	ND	ND
Gu Dong Bing	2010	Raw	12	Unknown	ND	ND	ND	ND	33.8
Yong Nian Jiu Jiu	2005	Raw	17	Yunxian country of Lincang City	ND	ND	ND	42.6	ND
Bu Lang Shan	2004	Raw	18	Bulang mountain	ND	ND	ND	ND	ND
Yi Wu	2001	Raw	21	Yiwu	ND	ND	ND	ND	ND
1999 Yi Chang Hao	1999	Raw	23	Unknown	ND	ND	ND	ND	ND
7582 Qing Cake	1995	Raw	27	Menghai County	ND	ND	ND	ND	ND
Zhu Tea	1992	Raw	30	Menghai County	ND	0.4	0.4	ND	ND
Yi Wu	1992	Raw	30	Yi Wu	ND	0.2	0.2	ND	ND
88 Qing Cake	1989	Raw	33	Menghai County	ND	ND	ND	ND	ND
8582 Qing Cake	1985	Raw	37	Unknown	ND	ND	ND	ND	ND
8582 Bozhi	1980	Raw	42	Menghai County	0.5	0.3	0.5	ND	ND
Wu Zhi Hong Yin	1950	Raw	72	Menghai County	0.5	0.1	0.3	ND	ND
Da Hong Yin	1950	Raw	72	Unknown	ND	ND	ND	ND	ND
Jing Chang Hao	1920	Raw	102	Jiang Cheng	0.8	0.1	0.5	ND	ND
Song Pin Hao	1900	Raw	122	Unknown	ND	ND	ND	ND	ND
Yi Run	2020	Ripened	2	Unknown	ND	ND	ND	28.0	ND
Bu Lang Shan	2020	Ripened	2	Bulang mountain	ND	ND	ND	ND	ND
Nan Nuo	2020	Ripened	2	Nannuo mountain	ND	ND	ND	ND	ND
Meng Song	2020	Ripened	2	Mengsong	ND	ND	ND	ND	ND
Bang Dong	2020	Ripened	2	Bangdong Village	ND	ND	ND	50.3	ND
Jing Mai	2020	Ripened	2	Jing Mai	ND	ND	ND	ND	ND
Lin Cang Yun Xian	2021	Ripened	1	Yunxian country of Lincang City	ND	ND	ND	ND	ND
Gong Ming	2005	Ripened	17	Dian Lake	ND	ND	ND	25.4	ND

AFB1: aflatoxin B_1_; AFG1: aflatoxin G_1_; AFG2: aflatoxin G_2_; DON: deoxynivalenol; ND: not detected.

## Data Availability

The datasets generated during the current study are contained within this article and the Supplementary Materials.

## Supplementary Materials

The following supporting information can be downloaded at https://www.mdpi.com/article/10.3390/molecules28196883/s1: Figure S1. MRM chromatograms of aflatoxin B_1_ (A) and fenazaquin (B) with 0.1% formic acid, 0.1% acetic acid and 5, 10 and 15 mM ammonia acetate as mobile phase modifiers. Figure S2. Representative UPLC-MS/MS chromatograms obtained from a blank Pu-erh tea sample. Figure S3. Representative UPLC-MS/MS chromatograms obtained from a standard in the solvent mixture.
