# Supplementary material for: Simultaneous Determination of Pesticide Residues and Mycotoxins in Storage Pu-erh Tea Using Ultra-High-Performance Liquid Chromatography Coupled with Tandem Mass Spectrometry
Source: Molecules. 2023 Sep 30;28(19):6883. doi: 10.3390/molecules28196883 (PMC10574668; doi:10.3390/molecules28196883)
Supplement: Supplementary file 1 [file molecules-28-06883-s001.zip › molecules-2548460-supplementary.pdf]

Article

Simultaneous Determination of Pesticide Residues and Mycotoxins in Storage Pu-erh Tea Using Ultra-High-Performance Liquid Chromatography Coupled with Tandem Mass Spectrometry

Siu Leung Chau [1], Aihua Zhao [2], Wei Jia [1] and Lu Wang [1,*]

[1] School of Chinese Medicine, Hong Kong Baptist University, Kowloon Tong, Hong Kong, China; slchau@hkbu.edu.hk (S.L.C.); weijia1@hkbu.edu.hk (W.J.)
[2] Shanghai Sixth People's Hospital Affiliated to Shanghai Jiao Tong University School of Medicine, Shanghai 200023, China; zhah@sjtu.edu.cn
* Correspondence: luwang1@hkbu.edu.hk

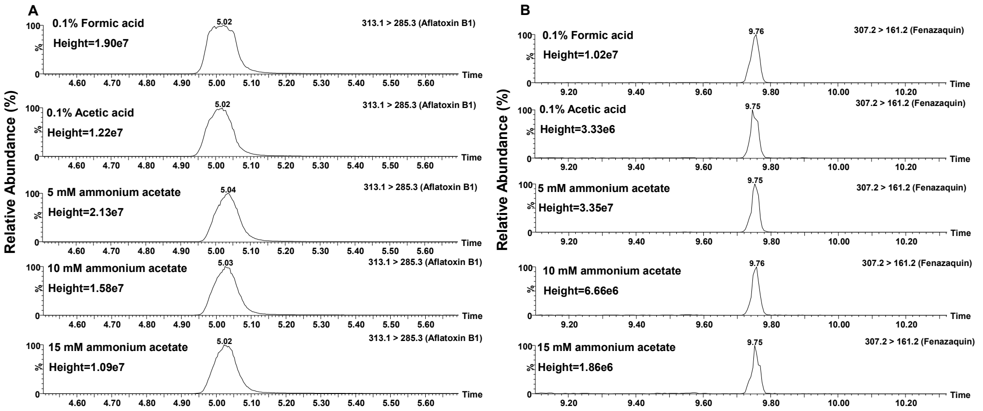

Figure S1. MRM chromatograms of aflatoxin B_1 (A) and fenazaquin (B) with 0.1% formic acid, 0.1% acetic acid and 5, 10 and 15 mM ammonia acetate as mobile phase modifiers.

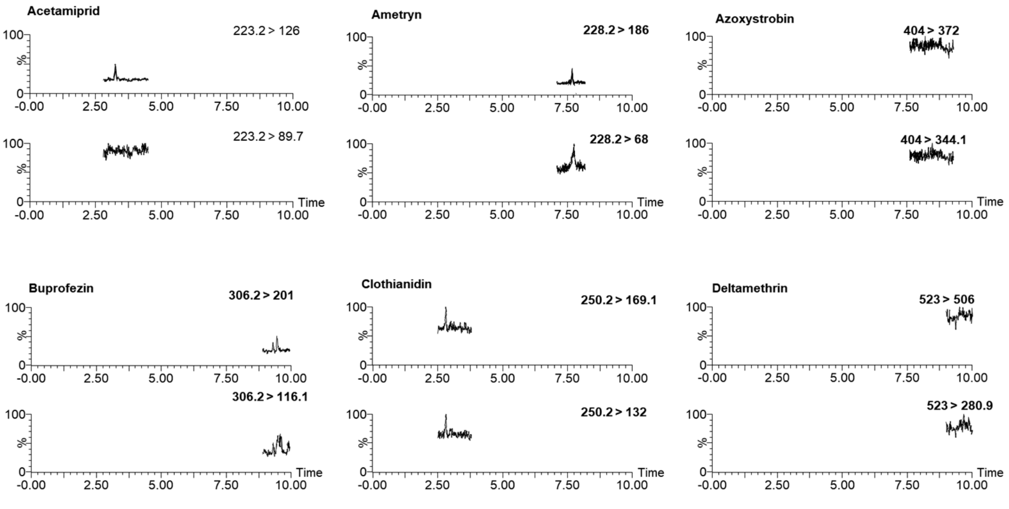

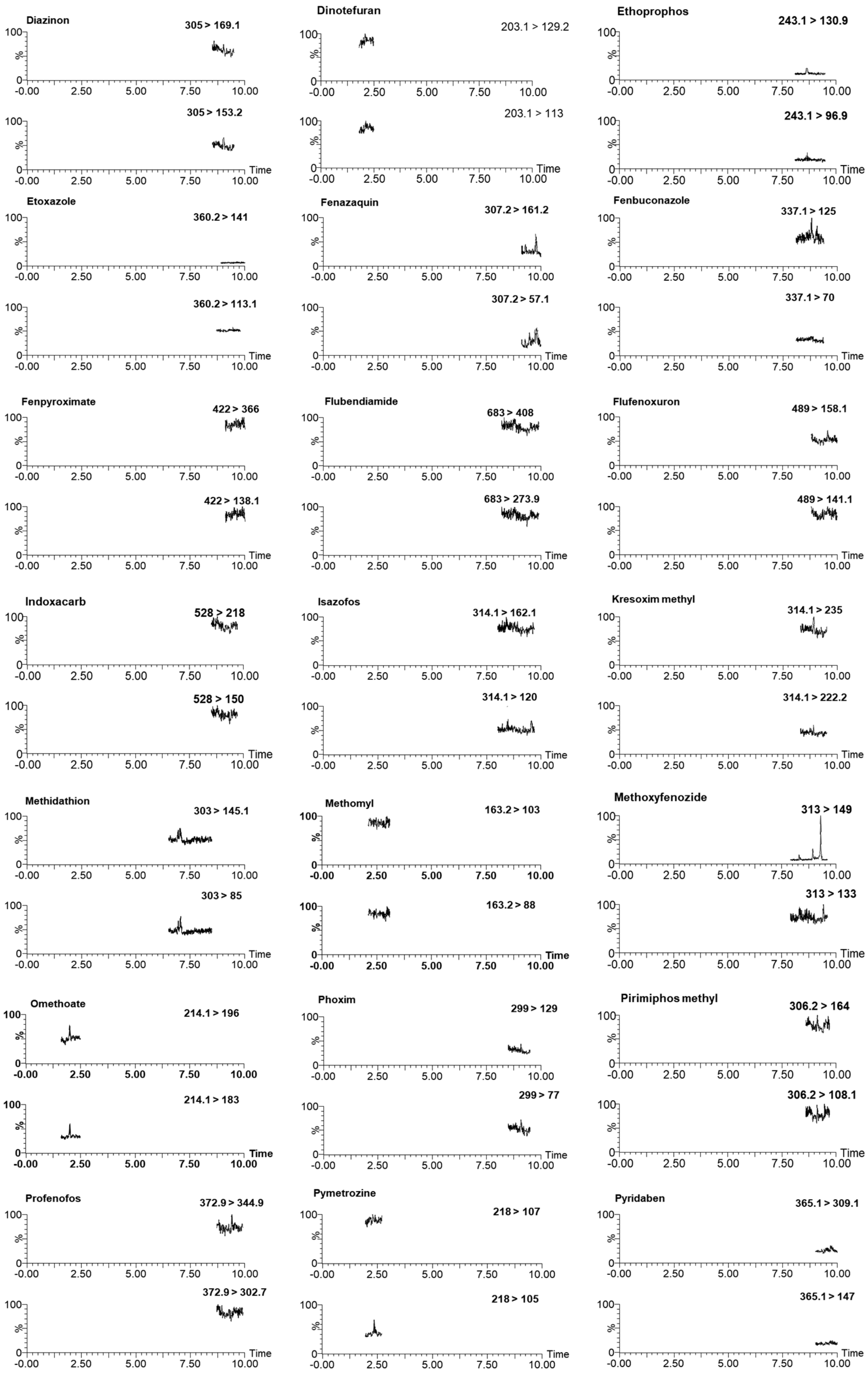
Diazinon
305 > 169.1
305 > 153.2
Dinotefuran
203.1 > 129.2
203.1 > 113
Ethoprophos
243.1 > 130.9
243.1 > 96.9
Etoxazole
360.2 > 141
360.2 > 113.1
Fenazaquin
307.2 > 161.2
307.2 > 57.1
Fenbuconazole
337.1 > 125
337.1 > 70
Fenpyroximate
422 > 366
422 > 138.1
Flubendiamide
683 > 408
683 > 273.9
Flufenoxuron
489 > 158.1
489 > 141.1
Indoxacarb
528 > 218
528 > 150
Isazofos
314.1 > 162.1
314.1 > 120
Kresoxim methyl
314.1 > 235
314.1 > 222.2
Methidathion
303 > 145.1
303 > 85
Methomyl
163.2 > 103
163.2 > 88
Methoxyfenozide
313 > 149
313 > 133
Omethoate
214.1 > 196
214.1 > 183
Phoxim
299 > 129
299 > 77
Pirimiphos methyl
306.2 > 164
306.2 > 108.1
Profenofos
372.9 > 344.9
372.9 > 302.7
Pymetrozine
218 > 107
218 > 105
Pyridaben
365.1 > 309.1
365.1 > 147
%
Time
-0.00
2.50
5.00
7.50
10.00

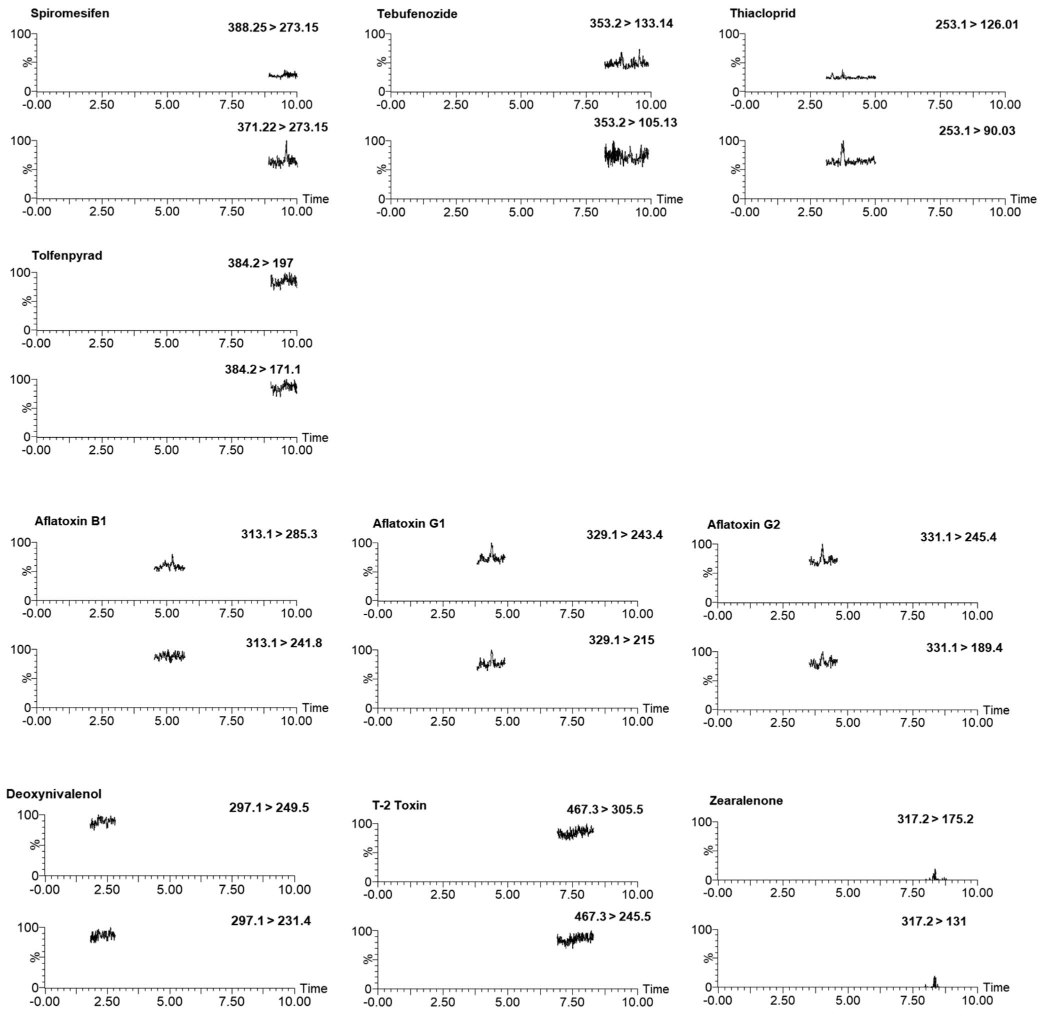

Figure S2. Representative UPLC-MS/MS chromatograms obtained from a blank Pu-erh tea sample.

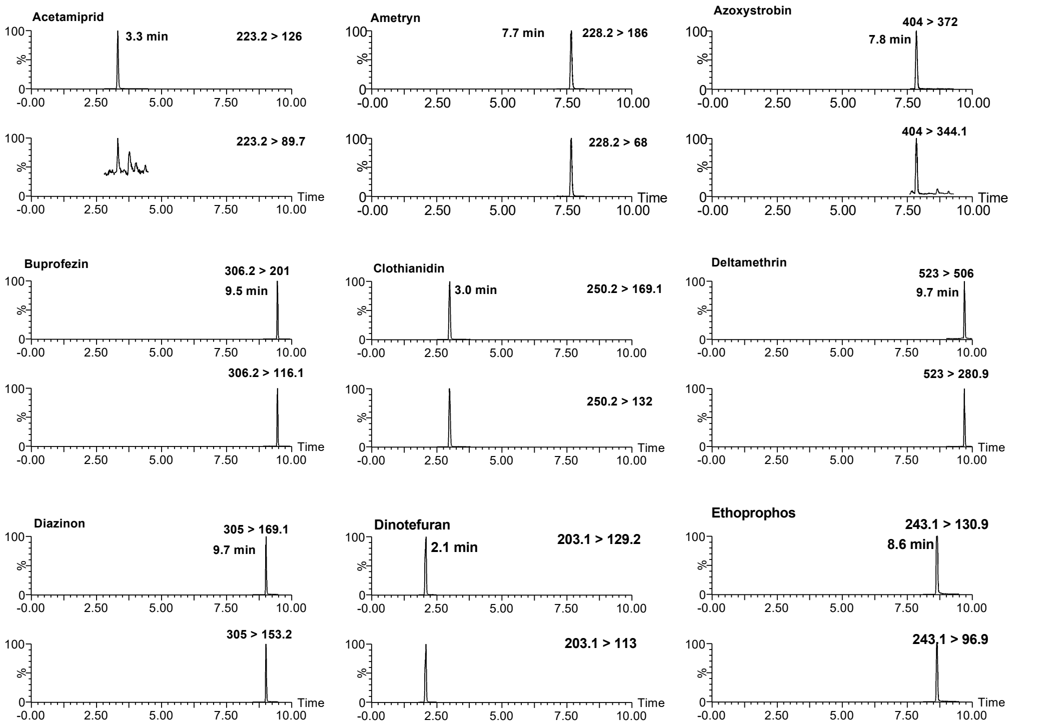

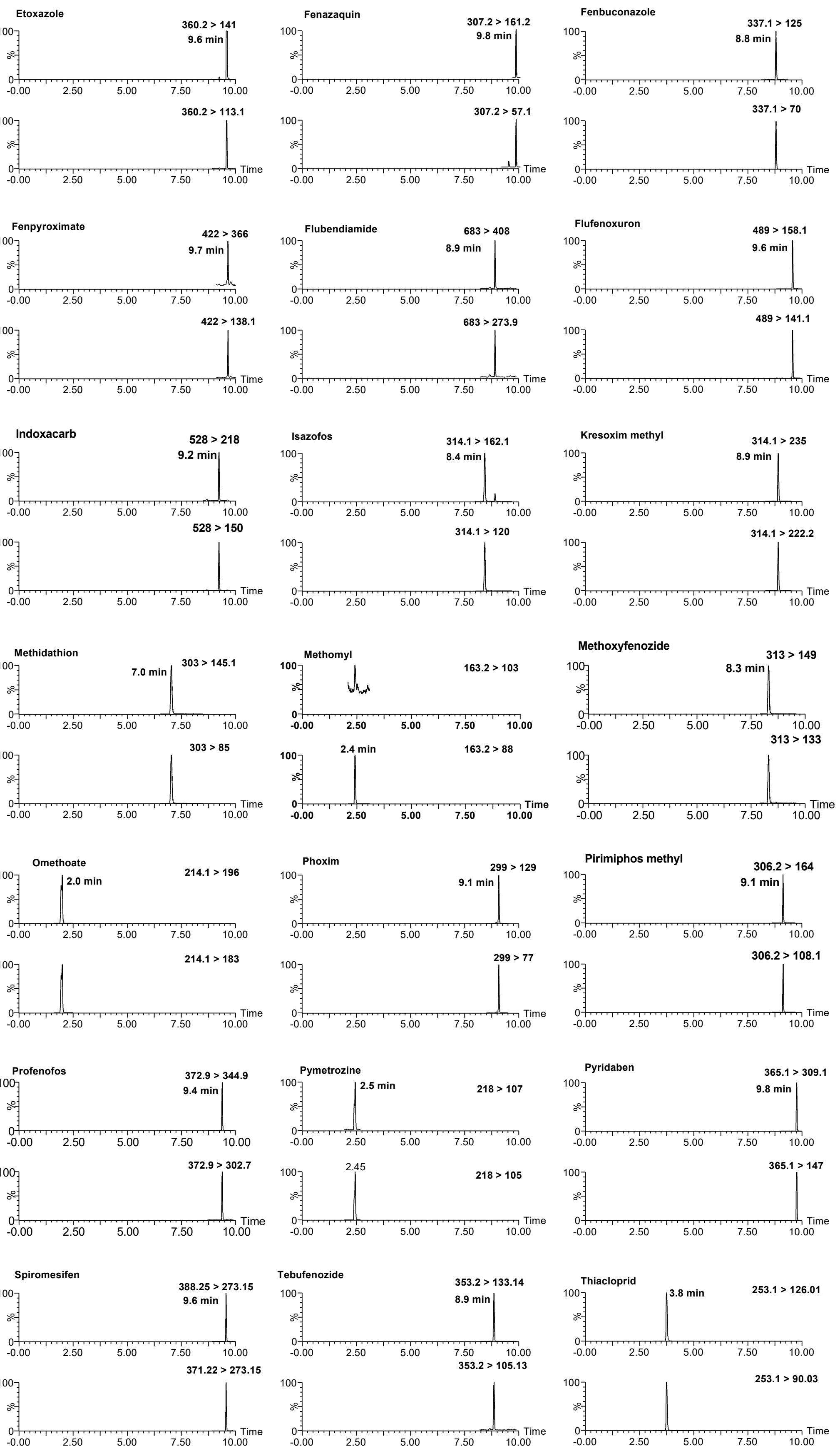
Etoxazole
360.2 > 141
9.6 min
360.2 > 113.1
Fenazaquin
307.2 > 161.2
9.8 min
307.2 > 57.1
Fenbuconazole
337.1 > 125
8.8 min
337.1 > 70
Fenpyroximate
422 > 366
9.7 min
422 > 138.1
Flubendiamide
683 > 408
8.9 min
683 > 273.9
Flufenoxuron
489 > 158.1
9.6 min
489 > 141.1
Indoxacarb
528 > 218
9.2 min
528 > 150
Isazofos
314.1 > 162.1
8.4 min
314.1 > 120
Kresoxim methyl
314.1 > 235
8.9 min
314.1 > 222.2
Methidathion
303 > 145.1
7.0 min
303 > 85
Methomyl
163.2 > 103
2.4 min
163.2 > 88
Methoxyfenozide
313 > 149
8.3 min
313 > 133
Omethoate
214.1 > 196
2.0 min
214.1 > 183
Phoxim
299 > 129
9.1 min
299 > 77
Pirimiphos methyl
306.2 > 164
9.1 min
306.2 > 108.1
Profenofos
372.9 > 344.9
9.4 min
372.9 > 302.7
Pymetrozine
2.5 min
218 > 107
2.45
218 > 105
Pyridaben
365.1 > 309.1
9.8 min
365.1 > 147
Spiromesifen
388.25 > 273.15
9.6 min
371.22 > 273.15
Tebufenozide
353.2 > 133.14
8.9 min
353.2 > 105.13
Thiacloprid
3.8 min
253.1 > 126.01
253.1 > 90.03
100
%
0
-0.00
2.50
5.00
7.50
10.00
Time

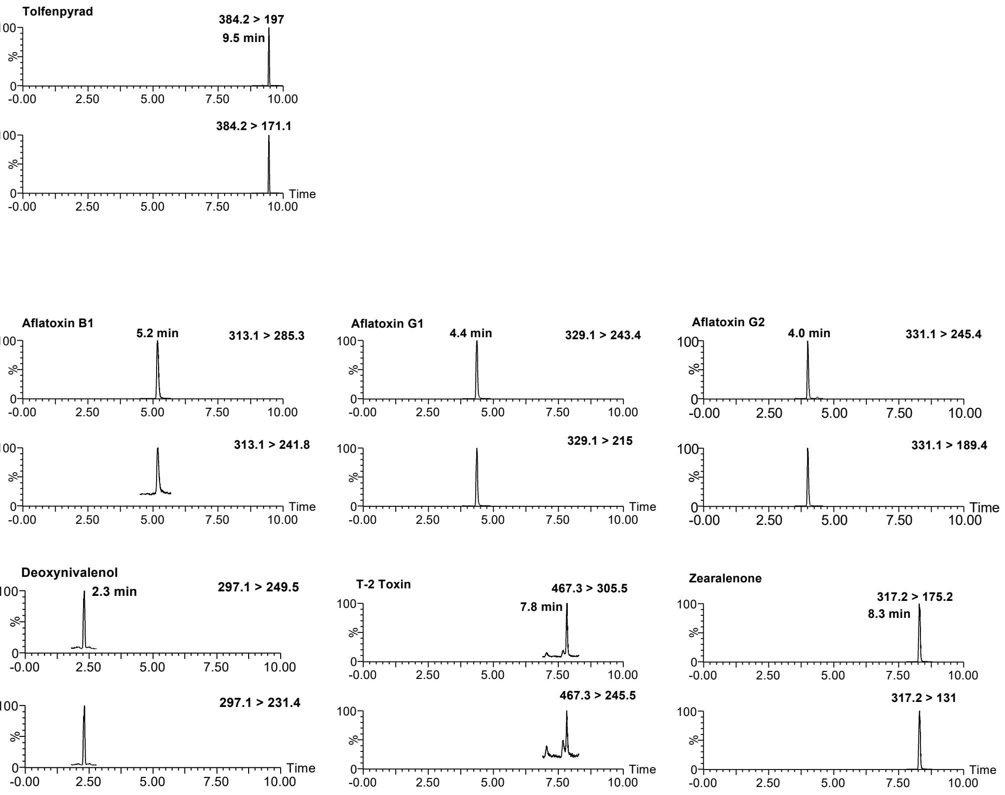

Figure S3. Representative UPLC-MS/MS chromatograms obtained from a standard in the solvent mixture.
